# Brain metabolic changes across King’s stages in amyotrophic lateral sclerosis: a ^18^F-2-fluoro-2-deoxy-d-glucose-positron emission tomography study

**DOI:** 10.1007/s00259-020-05053-w

**Published:** 2020-10-07

**Authors:** Antonio Canosa, Andrea Calvo, Cristina Moglia, Umberto Manera, Rosario Vasta, Francesca Di Pede, Sara Cabras, Davide Nardo, Vincenzo Arena, Maurizio Grassano, Fabrizio D’Ovidio, Koen Van Laere, Philip Van Damme, Marco Pagani, Adriano Chiò

**Affiliations:** 1 ALS Centre, “Rita Levi Montalcini” Department of Neuroscience, University of Turin, Via Cherasco 15, 10126 Turin, Italy; 2 Azienda Ospedaliero-Universitaria Città della Salute e della Scienza di Torino, Turin, Italy; 3 Neuroscience Institute of Turin (NIT), Turin, Italy; 4 MRC Cognition and Brain Sciences Unit, University of Cambridge, Cambridge, UK; 5 Positron Emission Tomography Centre AFFIDEA-IRMET S.p.A, Turin, Italy; 6 Department of Imaging and Pathology, Nuclear Medicine and Molecular Imaging, KU Leuven – University of Leuven, Leuven, Belgium; 7 Division of Nuclear Medicine, University Hospitals Leuven, Leuven, Belgium; 8 Department of Neurosciences, Experimental Neurology, and Leuven Brain Institute (LBI), KU Leuven – University of Leuven, Leuven, Belgium; 9 VIB, Center for Brain & Disease Research, Laboratory of Neurobiology, Leuven, Belgium; 10 Department of Neurology, University Hospitals Leuven, Leuven, Belgium; 11 Institute of Cognitive Sciences and Technologies, C.N.R., Rome, Italy; 12 Department of Medical Radiation Physics and Nuclear Medicine, Karolinska University Hospital, Stockholm, Sweden

**Keywords:** Amyotrophic lateral sclerosis, ^18^F-FDG-PET, King’s staging system

## Abstract

**Purpose:**

To assess the brain metabolic correlates of the different regional extent of ALS, evaluated with the King’s staging system, using brain ^18^F-2-fluoro-2-deoxy-d-glucose-PET (^18^F-FDG-PET).

**Methods:**

Three hundred ninety ALS cases with King’s stages 1, 2, and 3 (*n* = 390), i.e., involvement of 1, 2, and 3 body regions respectively, underwent brain ^18^F-FDG-PET at diagnosis. King’s stage at PET was derived from ALSFRS-R and was regressed out against whole-brain metabolism in the whole sample. The full factorial design confirmed the hypothesis that differences among groups (King’s 1, King’s 2, King’s 3, and 40 healthy controls (HC)) existed overall. Comparisons among stages and between each group and HC were performed. We included age at PET and sex as covariates.

**Results:**

Brain metabolism was inversely correlated with stage in medial frontal gyrus bilaterally, and right precentral and postcentral gyri. The full factorial design resulted in a significant main effect of groups. There was no significant difference between stages 1 and 2. Comparing stage 3 to stage 1+2, a significant relative hypometabolism was highlighted in the former in the left precentral and medial frontal gyri, and in the right medial frontal, postcentral, precentral, and middle frontal gyri. The comparisons between each group and HC showed the extension of frontal metabolic changes from stage 1 to stage 3, with the larger metabolic gap between stages 2 and 3.

**Conclusions:**

Our findings support the hypothesis that in ALS, the propagation of neurodegeneration follows a corticofugal, regional ordered pattern, extending from the motor cortex to posterior and anterior regions.

**Electronic supplementary material:**

The online version of this article (10.1007/s00259-020-05053-w) contains supplementary material, which is available to authorized users.

## Introduction

Amyotrophic lateral sclerosis (ALS) is a neurodegenerative disease affecting upper and lower motor neurons, leading to muscle weakness and wasting that progressively spreads across body regions. In about 50% of cases, also prefrontal regions are involved, causing various degrees of cognitive impairment of the frontotemporal type [1]. Death usually occurs within 2–5 years due to respiratory failure [2]. It has been proposed that ALS pathology disseminates in a regional ordered sequence, following a cortico-efferent spreading model [3].

In recent years, King’s staging system has been proposed for ALS, mainly based on the extension of the disease across body regions [4]. It defines the following stages: 1, symptom onset (involvement of the first region); 2A, diagnosis; 2B, involvement of a second region; 3, involvement of a third region; 4A, need for gastrostomy; 4B, need for respiratory support (non-invasive ventilation). Central nervous system region involvement was defined by the presence of weakness, wasting, spasticity, dysphagia, or dysarthria. Regions were defined in the same way as for the El Escorial criteria [5]. Afterwards, an algorithm based on ALSFRS-R has been proposed to estimate King’s stages [6].

Published data about the neuroimaging correlates of King’s stages are limited to few magnetic resonance imaging (MRI) studies based on small samples [7–9]. The aim of this study was to assess the brain metabolic correlates of a different regional extent of ALS, evaluated according to King’s staging system, using the brain ^18^F-2-fluoro-2-deoxy-d-glucose-PET (^18^F-FDG-PET) since it is a measure of neuronal injury and degeneration in vivo [10].

## Materials and methods

### Study participants

Patients diagnosed with definite, probable, and probable laboratory-supported ALS according to El Escorial revised diagnostic criteria [5], who underwent brain ^18^F-FDG-PET at diagnosis between 2008 and 2015 at the ALS Centre of Turin (“Rita Levi Montalcini” Department of Neuroscience, University of Turin, Turin, Italy) were considered eligible for the study (*n* = 406). The present study includes patients whose brain ^18^F-FDG-PET scan was included in the analyses performed in previous publications.

Forty healthy controls (HC) were included. Subjects who were referred to the PET center for suspected lung cancer but in whom no oncologic disease was detected with ^18^F-FDG-PET/CT and who had a normal neurological assessment were considered eligible as controls. Otherwise, major systemic illnesses, major vision disturbances, psychiatric illnesses, and diseases affecting brain functioning and metabolism represented exclusion criteria.

### Genetic analysis

All patients underwent genetic analysis for *C9ORF72*, *SOD1*, *TARDBP*, and *FUS* genes. All the coding exons and 50 bp of the flanking intron-exon boundaries of *SOD1*, of exon 6 of *TARDBP*, and of exons 14 and 15 of *FUS* have been PCR amplified, sequenced using the BigDye Terminator v3.1 sequencing kit (Applied Biosystems Inc.), and run on an ABIPrism 3500 genetic analyzer. These exons were selected as the vast majority of known pathogenic variants are known to lie within these mutational hotspots. A repeat-primed PCR assay was used to screen for the presence of the GGGGCC hexanucleotide expansion in the first intron of *C9ORF72*.

### ^18^F-FDG-PET acquisition

^18^F-FDG-PET was performed according to published guidelines [11]. Patients fasted at least 6 h before the exam. Blood glucose was < 7.2 mmol/l in all cases before the procedure. After a 20-min rest, about 185 MBq of ^18^F-FDG was injected. The acquisition started 60 min after the injection. In the patient group, a whole-body scan was performed setting head first. In the control group, a separate brain scan was performed after the whole-body one with a time difference of 15 min. The ^18^F-FDG-PET acquisition procedure was performed in the same environmental conditions in patients and controls, according to published guidelines [11]. PET/CT scans were performed on a Discovery ST-E System (General Electric). Brain CT (slice thickness of 3.75 mm, 140 kV, 60–80 mAs) and PET scan were sequentially acquired, the former being used for attenuation correction of PET data. The PET images were reconstructed with 4 iterations and 28 subsets with an initial voxel size of 2.34 × 2.34 × 2.00 mm and data were collected in 128 × 128 matrices.

### Assessment of King’s stage

King’s staging is based on the spreading of motor symptoms in three different body regions (bulbar, upper limbs, and lower limbs) and on the use of non-invasive ventilation (NIV) and enteral nutrition. We used the algorithm proposed by Balendra et al. [6] to calculate King’s stage from ALSFRS-R. The bulbar region was considered involved if a patient lost any points on any of the three items regarding speech, salivation, or swallowing (items 1, 2, and 3). The upper limb region was considered involved if a patient lost any points on either of the two items regarding handwriting and ability to cut food and handle utensils (items 4 and 5A). The lower limb region was considered involved if a patient lost any points on the item regarding walking (item 8). The presence of gastrostomy was confirmed by the assessment of item 5B (evaluation of the ability to manipulate fastenings if a patient has a gastrostomy) rather than item 5A (answered by patients without gastrostomy). If a subject scored 0 points on question 10 (indicating that the patient has significant difficulty with dyspnea and is considering using mechanical respiratory support) or less than 4 points on question 12 (dropping any points on this question indicates that Bi-level airway pressure ventilation is being used), this indicated that the patient was using NIV. We classified patients according to the following five stages of King’s staging system: 1, one region involved; 2, two regions involved; 3, three regions involved; 4A, patient needs gastrostomy; 4B, patient needs NIV.

Since our aim was to evaluate the metabolic changes related to the different regional extent of ALS, we focused on patients classified as King’s stages 1, 2, and 3 (*n* = 390). Indeed, stages 1, 2, and 3 correspond to involvement of 1, 2, and 3 body regions respectively, whereas stages 4A and 4B correspond to the reaching of milestones related to functional impairment (i.e., gastrostomy and NIV).

### Statistical analysis

The demographic and clinical characteristics of patient groups (King’s stages 1, 2, and 3) and HC were compared as follows. The *χ*^2^ test was employed for categorical variables. The analysis of variance (ANOVA) or the Kruskal-Wallis test was used for quantitative, continuous variables. The homogeneity assumption needed for ANOVA was evaluated through Levene’s test. In the case of significant Levene’s test, the Kruskal-Wallis test was employed instead of ANOVA.

SPM12 implemented in Matlab R2018b (MathWorks, Natick, MA, USA) was used for image spatial normalization to a customized brain ^18^F-FDG-PET template [12]. Intensity normalization was performed using the 0.8 default SPM value of gray matter threshold and images were subsequently smoothed with a 10-mm filter and submitted to statistical analysis.

In our sample (*n* = 390), King’s stage (1, 2, 3) was regressed out against whole-brain metabolism. The SPM12 multiple regression routine was implemented with age at PET and sex as covariates and the height threshold was set at *p* < 0.001 (*p* < 0.05 FWE corrected at cluster level). We used the full factorial design as implemented in SPM12 to test the hypothesis that differences among groups (King’s 1, King’s 2, King’s 3, HC) exist overall (i.e., main effect of groups). In case the hypothesis was confirmed, comparisons between groups defined according to King’s stage were performed through the two-sample *t* test model of SPM12. For both analyses, age at PET and sex were used as covariates and the height threshold was set at *p* < 0.001 (*p* < 0.05 FWE corrected at cluster level). Although the scope of the study was the assessment of metabolic differences across King’s stages, for a more exhaustive characterization of patient metabolic state, each group defined according to King’s stage was compared with HC, through the two-sample *t* test model of SPM12, with age at PET and sex as covariates, setting the height threshold at *p* < 0.0001 (*p* < 0.05 FWE corrected at cluster level). In all the analyses, only clusters containing > 125 contiguous voxels were considered significant. Brodmann areas (BAs) were identified at a 0–2-mm range from the Talairach coordinates of the SPM output isocenters corrected by Talairach Client (http://www.talairach.org/index.html).

## Results

### Demographic and clinical data

Based on the ALSFRS-R at the time of PET, 165 patients (42.3%) were in King’s stage 1, 133 (34.1%) in stage 2, and 92 (23.6%) in stage 3. The demographic and clinical characteristics of patients belonging to the three groups and of the whole sample are reported in Table 1. We found a significant difference among the three groups for the site of onset (*p* < 0.001), ALSFRS-R total score (*p* < 0.001), and cognitive status as defined by diagnostic criteria published by Strong et al. [13] (*p* = 0.019). However, a bivariate correlation analysis did not show any correlation between the site of onset and King’s stage (*p* = 0.36). Regarding the difference in ALSFRS-R total score, this could be expected since King’s stage has been calculated based on the ALSFRS-R score according to the algorithm published by Balendra et al. [6]. As for the differences in cognitive status, these confirm a previous study showing that cognitive impairment in ALS tends to be more severe as King’s clinical stage increases [14].

**Table 1:** Demographic and clinical characteristics of patients belonging to the three groups (King’s stages 1, 2, and 3) and of the whole sample. Data about the presence/absence of genetic mutations were available for 369 out of 390 patients. The neuropsychological assessment was available for 267 out of 390 patients: cognitive status was classified according to diagnostic criteria published by Strong et al. [13]

		King’s stage			*p* value	Total (*n* = 390)
		1 (*n* = 165)	2 (*n* = 133)	3 (*n* = 92)		
Sex	F (%)	69 (41.8%)	58 (43.6%)	52 (56.5%)	*p* = 0.062	179 (45.9%)
	M (%)	96 (58.2%)	75 (56.4%)	40 (43.5%)		211 (54.1%)
	Total	165	133	92		390
Age at PET, median (IQR)		64.2 (57.5–70.9)	64.0 (53.6–72.6)	67.1 (59.9–73.7)	*p* = 0.076	64.6 (56.6–71.9)
Age at diagnosis, median (IQR)		63.9 (57.9–70.3)	63.2 (53.2–72.0)	66.8 (59.5–73.4)	*p* = 0.096	64.42 (56.4–71.7)
Disease duration (months) at PET, median (IQR)		11.7 (7.9–16.9)	11.9 (9.2–16.5)	13.2 (8.7–20.6)	*p* = 0.449	12.1 (8.5–17.9)
Site of onset	Bulbar (%)	58 (35.2%)	28 (21.1%)	41 (44.6%)	*p* < 0.001	127 (32.6%)
	Spinal (%)	107 (64.8%)	105 (78.9%)	51 (55.4%)		263 (67.4%)
	Total	165	133	92		390
*C9orf72* repeat expansion	Negative (%)	146 (90.1%)	108 (89.3%)	81 (94.2%)	*p* = 0.447	335 (90.8%)
	Positive (%)	16 (9.9%)	13 (10.7%)	5 (5.8%)		34 (9.2%)
	Total	162	121	86		369
*SOD1* mutations	Negative (%)	157 (96.9%)	119 (98.3%)	84 (97.7%)	*p* = 0.750	360 (97.6%)
	Positive (%)	5 (3.1%)	2 (1.7%)	2 (2.3%)		9 (2.4%)
	Total	162	121	86		369
*TARDBP* mutations	Negative (%)	157 (96.9%)	117 (96.7%)	83 (96.5%)	*p* = 0.986	357 (96.7%)
	Positive (%)	5 (3.1%)	4 (3.3%)	3 (3.5%)		12 (3.3%)
	Total	162	121	86		369
*FUS* mutations	Negative (%)	161 (99.4%)	118 (97.5%)	86 (100%)	*p* = 0.185	365 (98.9%)
	Positive (%)	1 (0.6%)	3 (2.5%)	0 (0%)		4 (1.1%)
	Total	162	121	86		369
ALS FRS-R total score, median (IQR)		45 (43–46)	39 (37–42)	33.50 (30–37)	*p* < 0.001	41 (36–44)
Cognitive status	ALS-Cn (%)	70 (58.3%)	38 (44.7%)	26 (41.9%)	*p* = 0.019	134 (50.2%)
	ALS-bi (%)	21 (17.5%)	22 (25.9%)	16 (25.8%)		59 (22.1%)
	ALS-ci (%)	16 (13.3%)	13 (15.3%)	3 (4.8%)		32 (12.0%)
	ALS-cbi (%)	7 (5.8%)	8 (9.4%)	7 (11.3%)		22 (8.2%)
	ALS-FTD (%)	6 (5.0%)	4 (4.7%)	10 (16.1%)		20 (7.5%)
	Tot	120	85	62		267

*F*, female; *M*, male; *IQR*, interquartile range; *ALSFRS-R*, ALS Functional Rating Scale – Revised; *ALS-Cn*, ALS with normal cognition; *ALS-bi*, ALS with behavioral impairment; *ALS-ci*, ALS with cognitive impairment; *ALS-cbi*, ALS with cognitive and behavioral impairment; *ALS-FTD*, ALS with frontotemporal dementia

In the HC group, the median age was 66.5 years (interquartile range 55.0–72.0), and the male/female ratio was 2.64 (29/11). In the comparisons between each stage group and HC, we found no significant difference for age and sex distribution, with the exception of sex distribution between King’s stage 3 and HC (*p* = 0.002). Nevertheless, both age and sex were included as covariates in the analyses.

### ^18^F-FDG-PET data

**Fig. 1:**
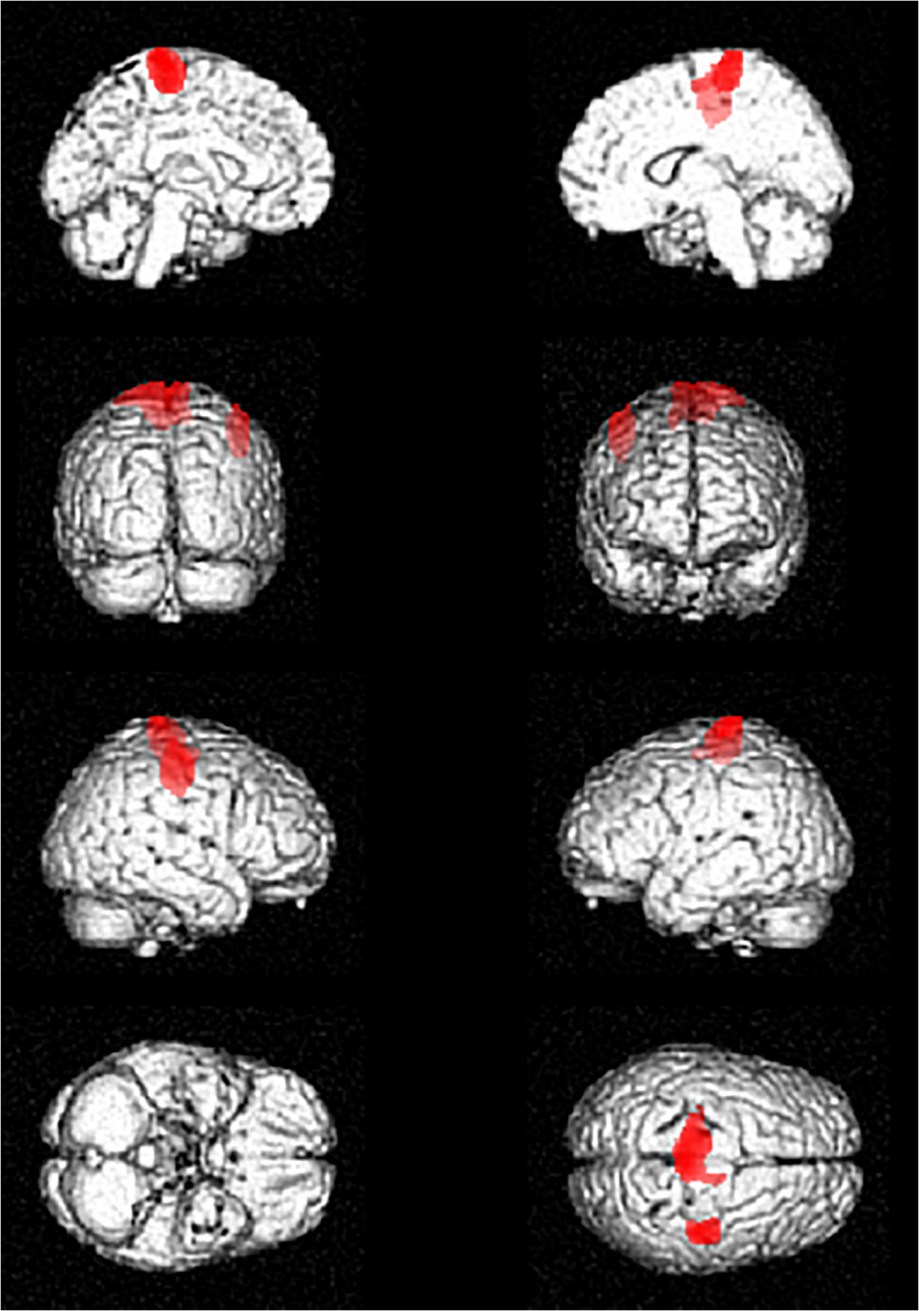
Glass brain rendering of multiple regression of King’s stage against whole-brain metabolism in the whole sample. The clusters showing a statistically significant negative correlation are projected on the brain surface

Using multiple regression analysis, we found an inverse correlation between brain metabolism and King’s stage (with stage 3 on the relatively hypometabolic side and stage 1 on the relatively hypermetabolic side). We identified clusters including the bilateral medial frontal gyrus (Brodmann area, BA, 6), right precentral gyrus (BA 4), and right postcentral gyrus (BA 2) (Fig. 1, Table 2). We did not identify any cluster with a positive correlation.

**Table 2:** Results of the negative correlation between whole-brain metabolism and King’s stage in the whole sample (*BA*, Brodmann area)

P (FWE-corr)	Cluster extent	Z-score	Talairach coordinates			Lobe	Cortical region	BA
0.001	1298	4.91	− 10	− 24	71	Frontal	Left medial frontal gyrus	6
		3.68	12	− 11	56	Frontal	Right medial frontal gyrus	6
0.021	658	4.05	44	− 15	50	Frontal	Right precentral gyrus	4
		3.98	44	− 23	47	Parietal	Right postcentral gyrus	2

**Fig. 2:**
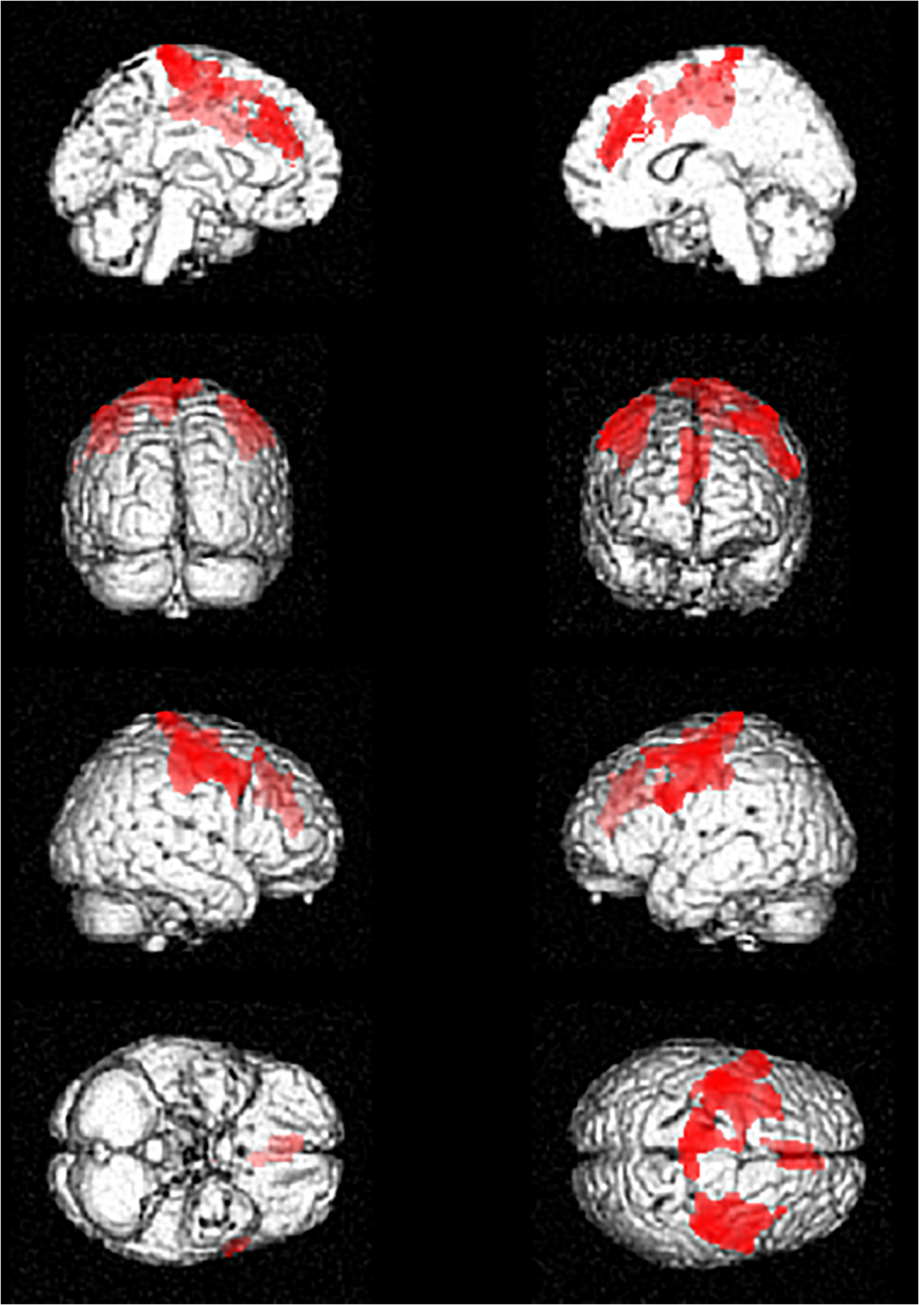
Glass brain rendering of the comparison: King’s stage 1+2 group versus King’s stage 3 group. The clusters showing a statistically significant relative hypometabolism in the King’s stage 3 group as compared with the King’s stage 1+2 group are projected on the brain surface

The full factorial design resulted in a significant main effect of groups (Supplemental Fig. 1). We hence computed the post hoc comparisons between the four groups. Comparing King’s stage 1 and King’s stage 2 groups, we did not find any significant difference. Therefore, we merged such groups into King’s 1+2 group. The comparison between King’s 1+2 group and the King’s 3 group revealed a significant relative hypometabolism in King’s 3 group in clusters including left precentral and medial frontal cortex (BAs 4 and 6), and right medial frontal, postcentral, precentral, and middle frontal cortex (BAs 4, 6, 3, 9, and 8) (Fig. 2, Table 3). King’s 3 group did not show any cluster of relative hypermetabolism as compared with King’s 1+2 group.

**Table 3:** Clusters showing a statistically significant relative hypometabolism in King’s stage 3 group as compared with King’s stage 1+2 group (*BA*, Brodmann Area)

*p* (FWE-corr)	Cluster extent	Z-score	Talairach coordinates			Lobe	Cortical region	BA
0.000	3321	5.11	− 42	− 9	56	Frontal	Left precentral gyrus	4
		4.67	− 8	− 18	64	Frontal	Left precentral gyrus	6
		4.41	− 10	− 24	71	Frontal	Left medial frontal gyrus	6
0.000	2089	4.46	38	− 13	54	Frontal	Right precentral gyrus	4
		4.40	40	12	42	Frontal	Right middle frontal gyrus	6
		4.05	44	− 21	40	Parietal	Right postcentral gyrus	3
0.006	899	3.58	8	40	24	Frontal	Right medial frontal gyrus	9
		3.45	6	29	41	Frontal	Right medial frontal gyrus	8

In the comparison with HC, we identified two clusters of relative hypometabolism in patient groups. The former included occipito-temporo-parietal regions and did not show a clear pattern of extension along with the increase of the clinical stage. The latter included frontal regions and showed an extension from left to right hemisphere from King’s stage 1 to stage 2 and a marked bilateral extension in stage 3 (Supplemental Figures 2, 3, and 4). No clusters of relative hypermetabolism in patient groups as compared with HC were found.

## Discussion

In this study, we explored the ^18^F-FDG-PET correlates of ALS clinical stages according to King’s staging system. We focused on King’s stages 1, 2, and 3, corresponding to the spreading of motor impairment to 1, 2, and 3 body regions respectively. A full factorial analysis showed that group differences exist at a global level and overlap the ones found between groups and groups and controls.

In the multiple regression analysis, brain metabolism negatively correlated with King’s stage. We found a decreasing gradient of metabolism going from King’s stage 1 to King’s stage 3 in clusters including right precentral and postcentral gyrus (BAs 4 and 2) and bilateral medial frontal gyrus (BA 6). When performing group comparisons, no significant difference was detected between King’s stage 1 group and King’s stage 2 group. King’s stage 3 group showed relatively hypometabolic clusters when compared with King’s stage 1+2 group, including left precentral and medial frontal gyrus (BAs 4 and 6) and right precentral, middle frontal, postcentral, and medial frontal gyrus (BAs 4, 6, 3, 8, and 9). The comparisons between each stage group and HC resulted in agreement with such findings, showing the extension of frontal metabolic changes from stage 1 to stage 3, with the larger metabolic gap between stages 2 and 3. Posterior clusters, including occipital lobes in all comparisons, of relative hypometabolism in ALS patients as compared with HC have already been reported [15].

The corticospinal tracts (CST) originate from neurons mainly situated in BA 4, corresponding to the primary motor cortex. Nevertheless, CST fibers rise also from neurons situated in other cortical regions, including the premotor and supplementary motor cortex (BA 6), and primary somatosensory cortex (BAs 1, 2, and 3). Therefore, we identified a decreasing gradient of metabolism going from patients with King’s stage 1 to subjects with King’s stage 3 in cortical regions from which the corticospinal tracts originate. Furthermore, group comparisons suggested that the main metabolic gap is situated between stages 2 and 3. We detected no difference between subjects with King’s stages 1 and 2. A possible explanation is that patients showing the involvement of three body regions at diagnosis subtend a more rapid neurodegenerative process, involving the prefrontal cortex to a greater extent, as compared with patients with stages 1 and 2. Otherwise, the metabolic difference between stages 1 and 2 is probably under the threshold, being the neurodegenerative process slower and less extensive.

Clusters of relative hypometabolism in subjects with King’s stage 3 as compared with patients with stages 1 and 2 included BAs 4, 6, and 3 and extended towards anterior regions (BAs 8 and 9). In a previous study, we identified a decreasing gradient of metabolism going from ALS with normal cognition to ALS with FTD, through cases with intermediate cognitive deficits (ALS-Ci) in frontal clusters including BAs 8 and 9 [16]. Synapse degeneration in BA 9 has been reported as a strong predictor of cognitive impairment in ALS [17]. Our findings are in agreement with neuropathological data suggesting that phosphorylated TDP-43 (p-TDP-43) tends to spread from the motor cortex to prefrontal and postcentral regions via axonal pathways [3]. According to such staging of p-TDP-43 pathology in ALS, BAs 4 and 6 are involved in stage 1, while the extension to the prefrontal and postcentral cortex is reported in stage 3. The data of the present study seem to strengthen the findings of our previous population-based cross-sectional study on cognitive impairment across ALS clinical stages [14], suggesting that cognitive deficits tend to be more severe as the clinical stage increases. In the proposed staging of p-TDP-43 pathology [3], the neurodegenerative process was thought to spread along axonal pathways. Our results are in agreement with such hypothesis that is further strengthened by several MRI studies, aimed at tracking the spreading of the neurodegenerative process in vivo through diffusion tensor imaging (DTI) [18–20], and simulation models based on brain network analyses [21, 22].

A ^18^F-FDG-PET study [23] on 146 patients evaluated the possible correspondence between the key brain regions used in the assessment of Brettschneider’s neuropathological staging system [3] and metabolic patterns allowing in vivo staging of ALS. This study found that the post-mortem neuropathological stages corresponded to distinct metabolic patterns.

The cross-sectional design of our study may have limited our findings. Nevertheless, patients were tested early after diagnosis and the cognitive impairment at that time point likely reflects the speed of lesion spreading to non-motor cortical areas of the brain. Besides, we can assume that King’s stages come in succession along the disease course. Indeed, in a recent study using databases from two multicenter clinical trials, 725 patients were retrospectively staged through the trial course. The authors reported that no reversions to earlier disease stages occurred and that most people progressed to the consecutive stage [24]. In ALS, longitudinal studies on neuroimaging are challenging since disability worsens over time: patients may be unable to undergo follow-up scans due to severe motor impairment and/or the development of respiratory failure. A recent review [25] evaluated published studies employing neuroimaging to track the course of ALS but did not report any ^18^F-FDG-PET study.

Another possible limitation should be considered in the interpretation of the asymmetry of the metabolic clusters identified in the analyses, due to the lacking of full information about handedness. Indeed, such data were available only for a part of the patient sample and were unavailable for controls. A further possible limitation is the absence of partial volume effect correction for cortical atrophy. Unfortunately, MRI scans were not available for all subjects. However, studies employing voxel-based atrophy correction of resting glucose metabolism showed that metabolic measurements were relatively independent of brain atrophy [26].

On the other hand, our study increases the knowledge about the neuroimaging correlates of King’s stages, which is still limited to few MRI studies with small samples. In this field, a recent study using magnetic resonance (MR) high-angular resolution diffusion imaging (HARDI), suggested an early pattern of microstructural degeneration in ALS, mainly involving the CST and the corpus callosum [7]. Nevertheless, in such study, patients with early disease stages were not compared with cases with more advanced stages. A further study investigated connectivity alterations associated with different stages of ALS [8], employing magnetoencephalography and MRI. The authors suggested that modifications, in terms of increased connectedness of functional brain networks, are related to disease progression. These results further support the hypothesis of the multi-system involvement of brain networks in ALS. Finally, a longitudinal study [9] reported that King’s ALS disease stages estimated from the ALSFRS-R correlated with CST diffusion measures in *C9orf72* carriers with heterogeneous clinical presentations (asymptomatic subjects, and ALS, ALS-FTD, and bvFTD patients). Based on the limited number of mutation carriers in our series, we think that the possible impact of genetic mutations on brain metabolism should be investigated in further studies including a larger amount of carriers.

Another strength of this study is the contribution to the debate about the use of brain ^18^F-FDG-PET in ALS. Recently, a panel of experts, on behalf of the European Association of Nuclear Medicine (EANM) and the European Academy of Neurology (EAN), did not recommend the clinical use of brain ^18^F-FDG-PET even to study ALS-related brain dysfunction [27, 28]. We feel that the present data support the use of ^18^F-FDG-PET to study ALS-related brain changes across motor and cognitive regions in a research setting. Otherwise, in agreement with the EANM-EAN recommendations, its use in the clinical setting needs further studies, including the comparison with ALS mimic disorders, to achieve a reliable accuracy at the single-patient level.

To our knowledge, this is the first study evaluating the brain metabolic correlates of regional extent expressed as disease staging in ALS and includes the largest ALS series with ^18^F-FDG-PET assessment ever published. We found that, with the increase of King’s stage, there was a decrease in metabolism in motor areas, with progressive involvement of extramotor regions. Since ^18^F-FDG-PET is a marker of neurodegeneration in vivo [10] and is considered valuable in cross-sectionally evaluating the spread of lesions in ALS [29], our data are in keeping with the ALS neuropathological staging model supporting that neurodegeneration extends from the motor cortex to posterior and anterior regions, possibly via axonal pathways [3], and are in agreement with our population-based, cross-sectional data showing that cognitive impairment tends to be more severe as the clinical stage increases [14]. Longitudinal studies are indispensable for the correlation of brain metabolic alterations with the progressive clinical involvement of different body regions and the possible cognitive deterioration over time.

## Electronic supplementary material

Supplemental Figure 1 Glass brain rendering of the full factorial analysis including the following groups: King’s stage 1, King’s stage, 2, King’s stage 3, and healthy controls. The clusters showing a significant main effect of groups are projected on brain surface. (PNG 801 kb)

High Resolution Image (TIF 170 kb)

Supplemental Figure 2 Glass brain rendering of the comparison: King’s stage 1 versus healthy controls. The clusters showing a statistically significant relative hypometabolism in the King’s stage 1 group as compared to healthy controls are projected on brain surface. (PNG 768 kb)

High Resolution Image (TIF 161 kb)

Supplemental Figure 3 Glass brain rendering of the comparison: King’s stage 2 versus healthy controls. The clusters showing a statistically significant relative hypometabolism in the King’s stage 2 group as compared to healthy controls are projected on brain surface. (PNG 731 kb)

High Resolution Image (TIF 155 kb)

Supplemental Figure 4 Glass brain rendering of the comparison: King’s stage 3 versus healthy controls. The clusters showing a statistically significant relative hypometabolism in the King’s stage 3 group as compared to healthy controls are projected on brain surface. (PNG 1265 kb)

High Resolution Image (TIF 174 kb)

## Data Availability

Data will be available upon request by interested researchers.
