# Supplementary figures and images for: Brain metabolic changes across King’s stages in amyotrophic lateral sclerosis: a 18F-2-fluoro-2-deoxy-d-glucose-positron emission tomography study
Source: Eur J Nucl Med Mol Imaging. 2020 Oct 7;48(4):1124–33. doi: 10.1007/s00259-020-05053-w (PMC8041703; doi:10.1007/s00259-020-05053-w)

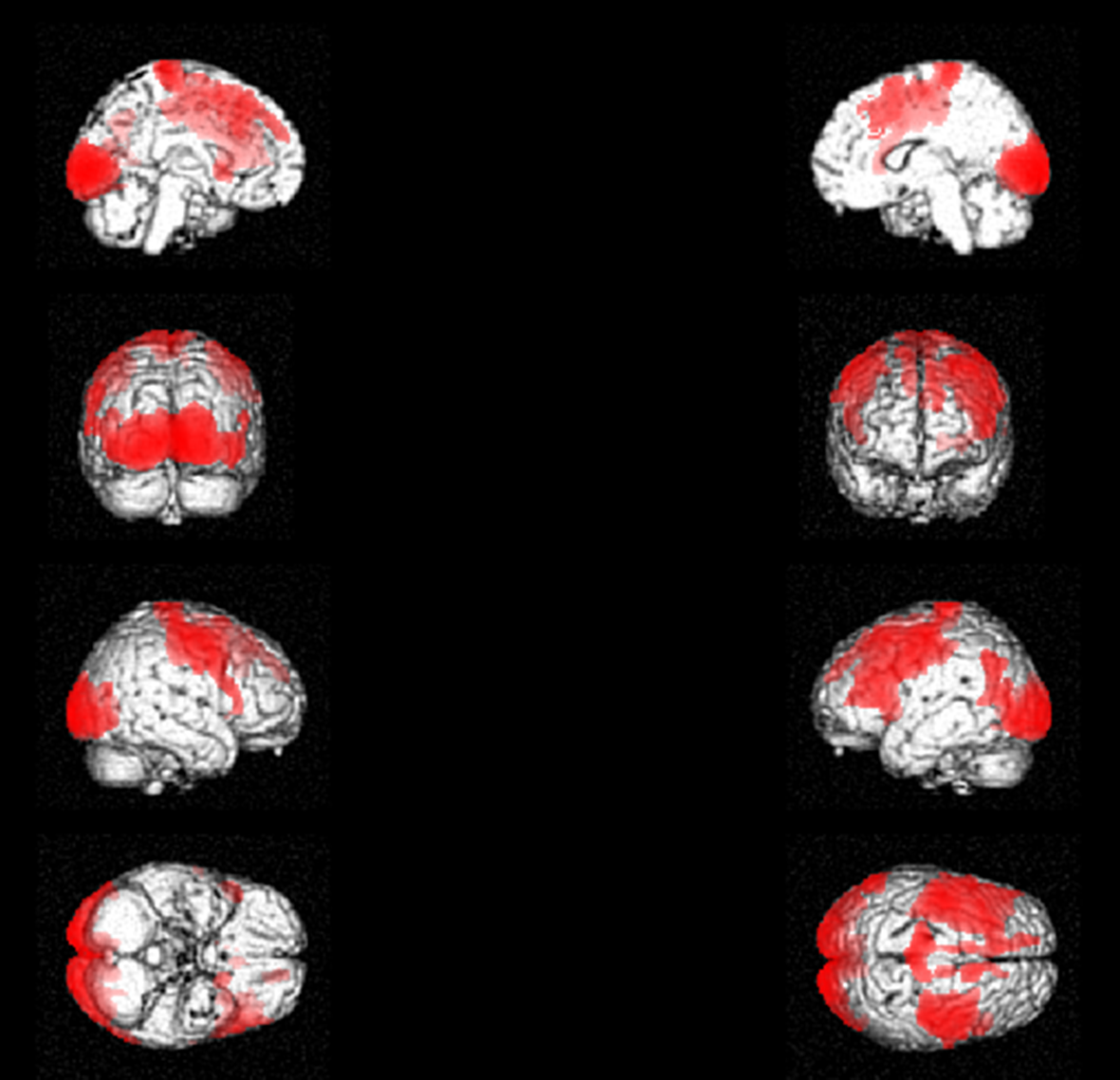

Supplement: Supplementary file 1 — Glass brain rendering of the full factorial analysis including the following groups: King’s stage 1, King’s stage, 2, King’s stage 3, and healthy controls. The clusters showing a significant main effect of groups are projected on brain surface. (PNG 801 kb) [file 259_2020_5053_Fig3_ESM.png]

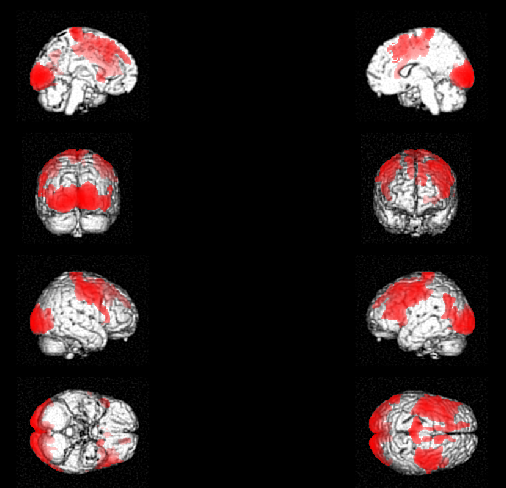

Supplement: Supplementary file 2 — High Resolution Image (TIF 170 kb) [file 259_2020_5053_MOESM1_ESM.tif]

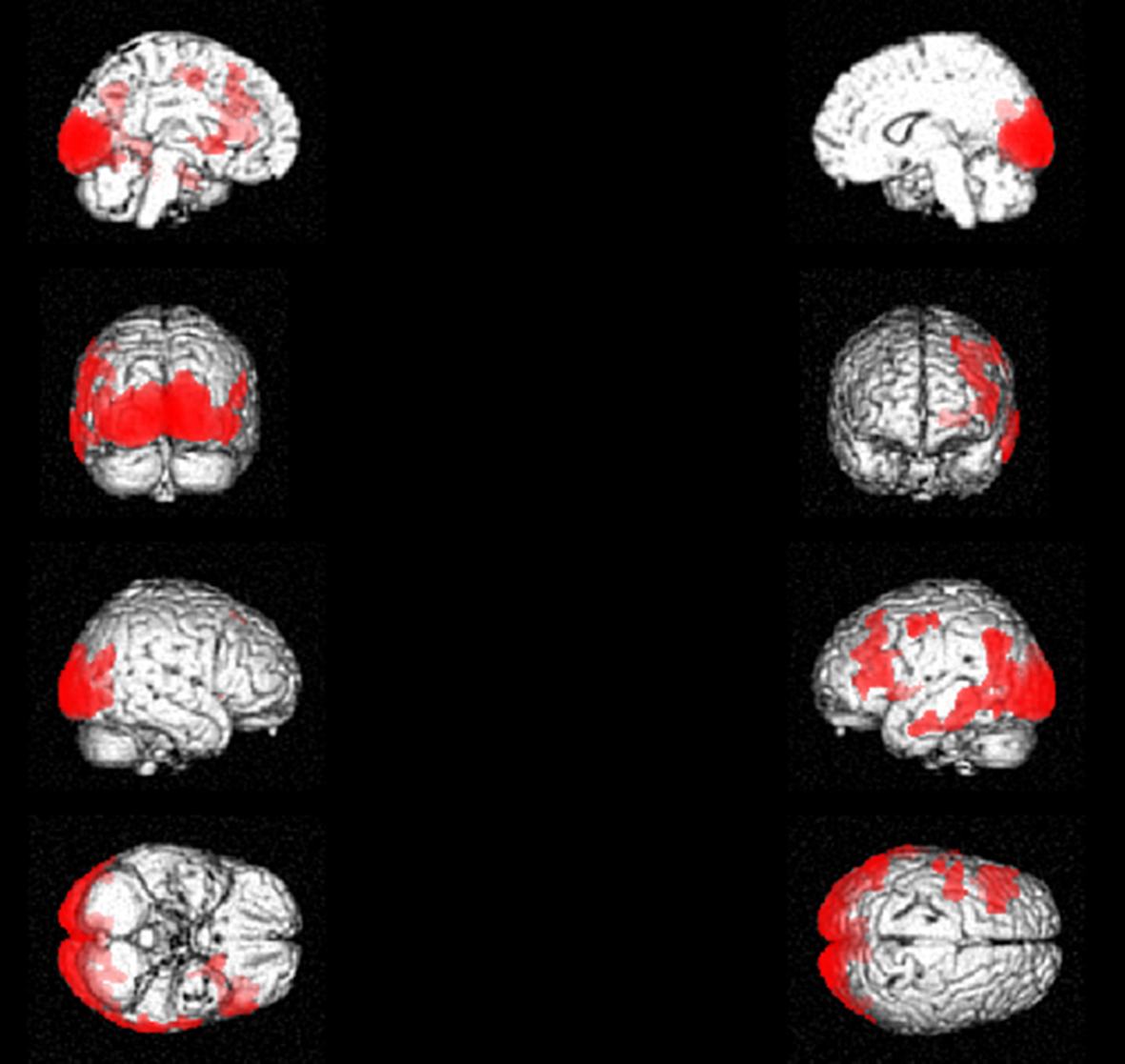

Supplement: Supplementary file 3 — Glass brain rendering of the comparison: King’s stage 1 versus healthy controls. The clusters showing a statistically significant relative hypometabolism in the King’s stage 1 group as compared to healthy controls are projected on brain surface. (PNG 768 kb) [file 259_2020_5053_Fig4_ESM.png]

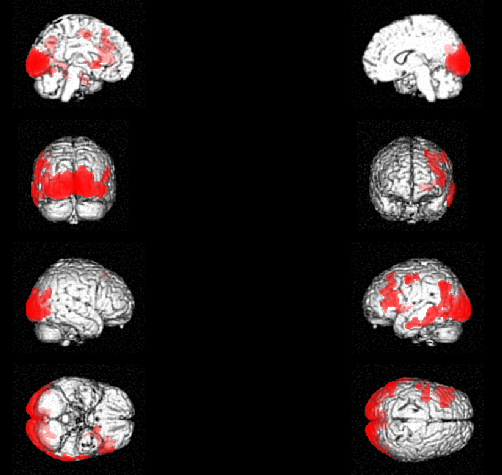

Supplement: Supplementary file 4 — High Resolution Image (TIF 161 kb) [file 259_2020_5053_MOESM2_ESM.tif]

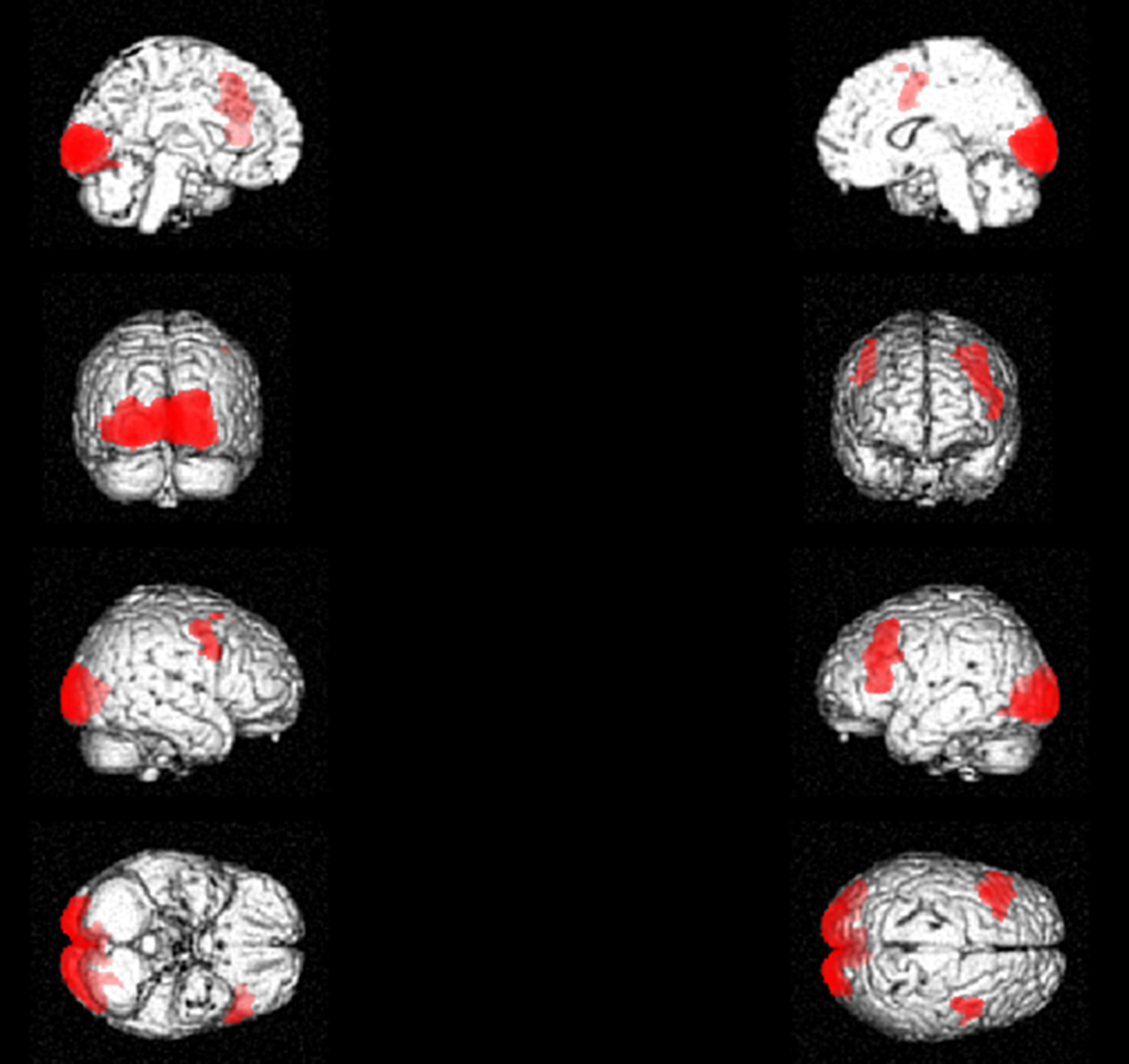

Supplement: Supplementary file 5 — Glass brain rendering of the comparison: King’s stage 2 versus healthy controls. The clusters showing a statistically significant relative hypometabolism in the King’s stage 2 group as compared to healthy controls are projected on brain surface. (PNG 731 kb) [file 259_2020_5053_Fig5_ESM.png]

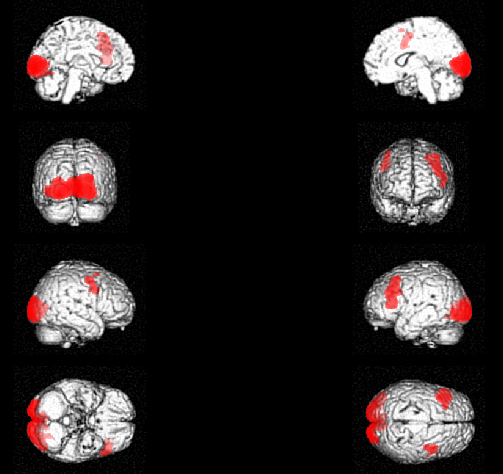

Supplement: Supplementary file 6 — High Resolution Image (TIF 155 kb) [file 259_2020_5053_MOESM3_ESM.tif]

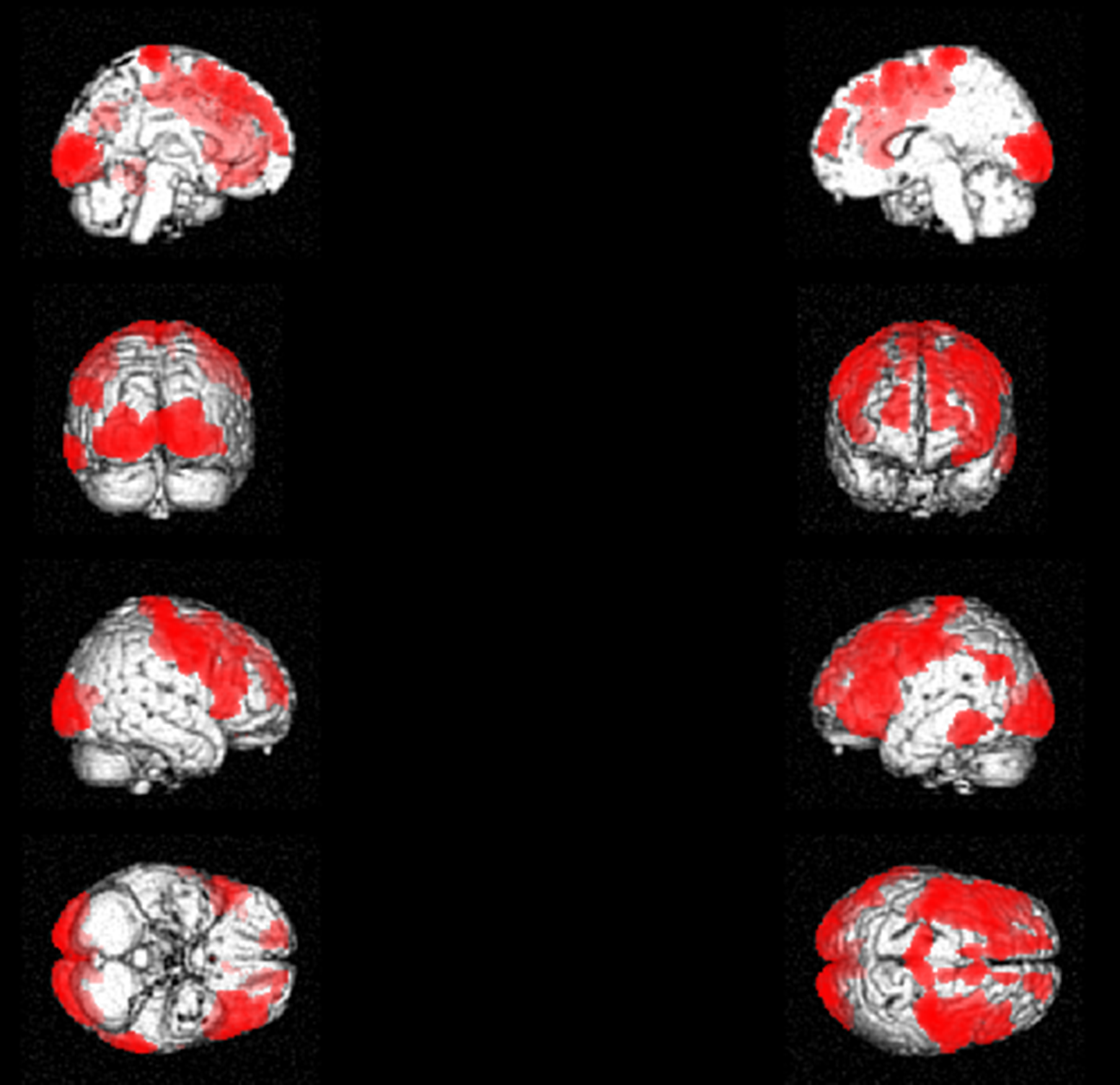

Supplement: Supplementary file 7 — Glass brain rendering of the comparison: King’s stage 3 versus healthy controls. The clusters showing a statistically significant relative hypometabolism in the King’s stage 3 group as compared to healthy controls are projected on brain surface. (PNG 1265 kb) [file 259_2020_5053_Fig6_ESM.png]

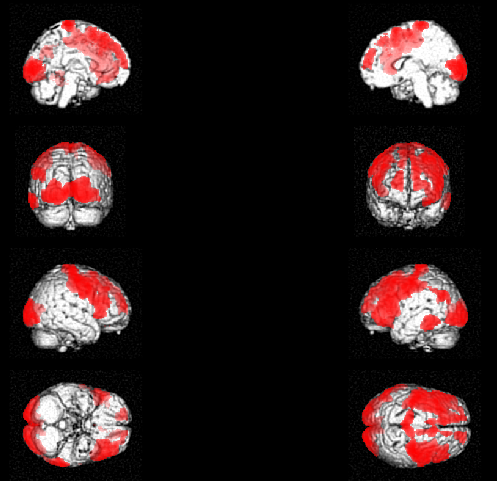

Supplement: Supplementary file 8 — High Resolution Image (TIF 174 kb) [file 259_2020_5053_MOESM4_ESM.tif]
